# An Australian perspective on clinical, economic and regulatory considerations in emerging nanoparticle therapies for infections

**DOI:** 10.1038/s44259-024-00070-3

**Published:** 2025-02-18

**Authors:** Benjamin W. Muir, Jennifer A. E. Payne, Jennifer H. Martin, Riley O’ Shea, Sarigama Rajesh, Lewis D. Blackman, Hsin-Hui Shen, Chad Heazlewood, Vipul Bansal, Branwen Morgan

**Affiliations:** 1 Commonwealth Scientific and Industrial Research Organisation, Clayton, VIC Australia; 2 University of Newcastle School of Medicine and Public Health, Callaghan New South Wales, Australia; 3 Monash University, Clayton, VIC Australia; 4 Sir Ian Potter NanoBioSensing Facility, RMIT University, Melbourne, VIC Australia

**Keywords:** Antibiotics, Antimicrobial resistance, Drug delivery, Nanoparticles, Drug regulation

## Abstract

Antimicrobial resistance (AMR) poses a growing global health threat. Nanomedicine, combined with drug repurposing, may help extend the effective lifespan of current and new antimicrobials. This review, presents an Australian perspective on nanotechnology-based therapies, highlighting scientific and clinical challenges. Early consideration of the potential barriers to market access may help to accelerate research translation, regulatory approval and patient access to nano-antimicrobial (NAM) drugs for resistant pathogens, not only in Australia, but globally.

## Introduction

Over the last five years, less than a dozen new antimicrobials (to treat fungal, parasitic, and bacterial infection) have been approved by the US Food and Drug Administration (FDA) and the European Medicines Authority (EMA)^1–3^. Continued growth in the antimicrobial clinical pipeline from R&D to clinical trials is essential, as deaths attributed and associated with bacterial antimicrobial resistance (AMR) continue to climb from the 2019 estimate of five million people^4^. Equally important is the decreased quality of life for people managing or surviving a drug-resistant infection. There is an urgent need to implement strategies to mitigate AMR, including accelerating drug development times and the of diversity infectious disease treatment options^5^. This includes repurposing existing medicines as antimicrobials and incorporating technological advancements in formulation and delivery mechanisms^6^.

One way of achieving this is through the development of nano-antimicrobial (NAM) therapies. The US FDA and the Australian Therapeutic Goods Administration (TGA) guidance defines nanotechnology products as those where the final product has been deliberately engineered at the 1–100 nm scale, up to one micron^7^ and where the attributes, such as pharmacodynamic and pharmacokinetics, of the product rely on the active material being in the nano-scale. NAMs are typically comprised of organic or inorganic materials, including lipids, polymers, peptides, proteins, metals, metal oxides, and a variety of composite materials^8,9^. NAMs can be coupled with other antimicrobial agents that can result in decreased dosing or combination regimens compared to the free agent^10–13^. NAMs are increasingly attractive when faced with rising rates of drug-resistant infections and a diminishing pipeline of new antimicrobials due to their inherent antimicrobial properties and ability to overcome mechanisms of resistance^12–16^. Due to their unique nature, scientific, economic, and regulatory requirements need to be specifically addressed for NAMs to ensure they can be successfully translated into clinical practice. Understanding these challenges may help accelerate the path to impact.

## Considerations and challenges for NAM development to address AMR

While antimicrobial NAMs are a potential niche opportunity, they will encounter the same market access and commercial viability issues faced by traditional antimicrobial counterparts^17–19^. Here we highlight a couple of key considerations when developing NAMs in Australia that are also relevant globally.

### Transitioning from R&D to clinical trials

One of the most challenging bottlenecks for new drugs occurs between pre-clinical and first-in-human clinical trials. Clinical trials conducted in Australia are accepted by key jurisdictions, including the US FDA and EMA and do not require US FDA Investigational New Drug (IND) application approval, which means trials can be initiated more quickly. There are also R&D tax incentives to attract investment, including clinical trials. However, it has remained difficult to recruit enough patients to statistically power antimicrobial studies in Australia due to the low infection numbers and dispersed population groups. Recruitment for clinical trials in remote and rural communities, and the inclusion of Indigenous peoples in Australia and other countries, remains even more problematic^20^. Public awareness and trust, as well as an understanding of the benefits that NAMs can impart, is also an important part of obtaining support for these clinical trials^21^. Additionally, antimicrobial clinical trials are disadvantaged by the lack of rapid specific diagnostics that could enable pathogen identification, enhance patient recruitment, and enrich clinical trial populations.

### Commercial viability in the face of rapid AMR and changes with push and pull incentives

There is a high cost of both time and money (up to US $2.6 bn and 10–15 years) to bring a new medicine to market, coupled with a limited period of exclusivity to profit from these discoveries^22^. For new antimicrobials, including NAMs, this model is not financially viable or competitive in comparison with other therapeutic areas^21^. It is, therefore, not surprising that many pharmaceutical companies have exited antimicrobial R&D in favour of a focus on non-communicable and chronic disease^3^. Small companies have filled the gap and are bringing new antimicrobials to market, including a few NAMs (see Supplementary Tables 1 and 2), helped by push incentives and targeted funding for antibiotic development initiatives such as Combating Antibiotic-Resistant Bacteria Biopharmaceutical Accelerator (CARB-X), the REPAIR Impact Fund, INCubator for Antibacterial Therapies in Europe (INCATE), and the AMR Action Fund. However, there is still a concerning low level of global AMR R&D, human capital, and expertise^19,21^.

Moreover, market success is not guaranteed, as post-approval expenses that are usually covered by high sales volumes are not feasible for antimicrobials. There is a balancing act between promoting and restricting the use of antimicrobials to limit the development of resistance and maximise the effective lifespans of the drug, which falls under antimicrobial stewardship. This is necessary because microbial pathogens are capable of rapidly developing and disseminating resistance, the rate of which is often correlated with the level of antibiotic use^17^. Since new antimicrobials are not regarded as lifesaving medicines, they generally have a low price expectation by patients. As drugs of ‘last resort’, they also have low sales volumes, and post-market entry costs that are higher than their revenue, which it is unsurprising for companies to be declared bankrupt, despite bringing a new antimicrobial to market^17^. However, innovative pricing and reimbursement mechanisms have been implemented in some countries, including the UK, and are expected to help reinvigorate the antimicrobial R&D pipeline^17,19,23^. To date, these ‘pull’ incentives, which delink the volume of antibiotic sales from profit, have not been implemented in Australia^2,3,5,17,22^.

## The potential of NAM therapies

Promising antimicrobial molecule candidates routinely fail clinical trials due to off-target toxicity effects and delivery challenges. These issues have been addressed by reformulating the product as NAMs and may be able to address these problems for future repurposing and new antimicrobials^2,3^. Using NAMs to alter the pharmacology of current and emerging therapies widens the potential range of AMR strategies^10^. The advantages of NAMs include: lower doses of antimicrobials to achieve the same effect; targeting of specific organisms or sites of infection, thereby reducing off-target cytotoxic effects; polytherapy approaches where two or more antimicrobial components are administered simultaneously to achieve an improved response; and the repurposing of existing drugs and new drugs that have issues such as poor solubility^6,10,24,25^.

### Improving targeting and delivery through nano-formulations

Clinical NAM therapies include dendrimer formulations for bacterial vaginosis (VivaGel), which was developed in Australia, and lipid NAM formulations that contain the antifungal Amphotericin B (AmBisome, Fungisome, Abelcet, Amphotec) or the antibiotic Amikacin (Arikayce)^10^. These lipid NAM formulations emphasise the benefits and bring into focus the regulatory and manufacturing requirements to ensure consistent NAM formulations.

Arikayce contains the antibiotic amikacin in liposomes composed of dipalmitoylphosphatidylcholine and cholesterol for the treatment of *Mycobacterium avium* complex lung disease^26^. This pathogen persists in biofilms or as an intracellular infection within macrophages, making it a difficult target for traditional systemic antibiotics. The NAM formulation is delivered through a bespoke nebuliser that ensures consistencies in the size and distribution of liposomes and the ratio of free:encapsulated drug, that are essential parameters for activity. It is designed to facilitate targeted drug delivery to the lungs while minimising systemic exposure. This reduces toxicity and the off-target effects that are observed with free-amikacin, and thus allows for longer term and higher dosing regimens. The NAM formulation also results in greater uptake of the encapsulated drug into macrophages by over five-fold, and increased biofilm penetration compared with the free drug in vivo, thereby making treatment more effective^26–29^.

Amphotericin B is a broad-spectrum antifungal that has acute infusion-related reactions and nephrotoxicity that often prevents complete course administration. Encapsulation of Amphotericin B in a range of different lipid NAM formulations (AmBisome, Fungisome, Abelcet, and Amphotec) has improved its efficacy and safety by reducing host toxicity through the controlled release and targeting of the drug to the fungal pathogen. For Amphotec, in vivo, results demonstrated a five-fold reduction in renal toxicity compared to free Amphotericin B^30^. The manufacturing process of AmBisome defines the required physical features of the liposomes. The same chemical compositions of the lipid nano-formulation with slight deviations in the methods of production result in significantly different toxicities and reduced efficacy. The failure to reproduce the exact structure has resulted in several generic AmBisome products being recalled by the FDA^31–33^. This difference in toxicity underscores the importance of regulation and testing across NAM products and batches.

### Overcoming AMR through nano-formulations

The mechanisms of action of NAMs cover a wide spectrum of antimicrobial activity^9,34^. There are examples of NAMs that can disrupt microbial cell walls and membranes, including the use of cationic NAMs and ‘nanoknives’ where the material's sharp pointy structures physically disrupt membranes. NAMs can produce reactive oxygen species (ROS), damaging intracellular components, including DNA, to block protein synthesis and cell division, and disrupt complex biofilms. They can allow inhibition and bactericidal mechanisms to operate in parallel and interfere with the mechanisms microbes use to circumvent DNA repair and develop AMR^8–10,21,25^.

NAMs can enable accessing infections typically harder to treat, including biofilms, and intracellular infections^35,36^. For example, biofilms are characterised by having a more acidic environment, allowing pH-sensitive liposomes to target and accumulate in biofilms. Additionally, NAM liposomes can enhance drug delivery to biofilms through the fusion of lipids with biofilm-dwelling bacterial membranes, while metal NAMs interfere with both quorum sensing and the extracellular matrix structure^24^. The activity against biofilms and intracellular uptake are features of the clinically available NAM Arikayce, where lipid encapsulation of antibiotic amikacin, enhances its activity against the intracellular pathogen *M. avium*^26–29^.

Pathogenic microorganisms have acquired numerous survival mechanisms enabling them to evade killing by antibiotics^9^. NAMs may circumvent traditional antibiotic resistance mechanisms, including cell wall thickening, expression of efflux pumps, and enzymatic degradation^9,10,24,25^. For example, lipid nanocarriers of various antibiotics markedly reduce the in vitro MIC of these drugs relative to the free drug control, against a wide range of bacterial species. This has been attributed to the fusogenicity of lipid nanoparticles, which aids membrane penetration, as well as the protection of the encapsulated drugs from enzymatic degradation^36,37^. Furthermore, new antimicrobial mechanisms imparted by NAMs themselves, such as cell membrane disruption by high aspect ratio carbon and metal nanomaterials (‘nanoknife’ mechanism), or cationic lipid and polymeric nanoparticles, enable broad-spectrum antimicrobial properties not currently overcome by existing bacterial resistance mechanisms^38^. Other previously unseen killing mechanisms include metal ion leaching, ROS production through photodynamic materials, and photothermal therapy, among others^39–43^. In summary, the unique mechanisms of action of nanoparticles, different from the traditional drug, make them ideal for combined therapies and overcoming multidrug-resistance mechanisms.

## Current NAM clinical pipeline

**Fig. 1:**
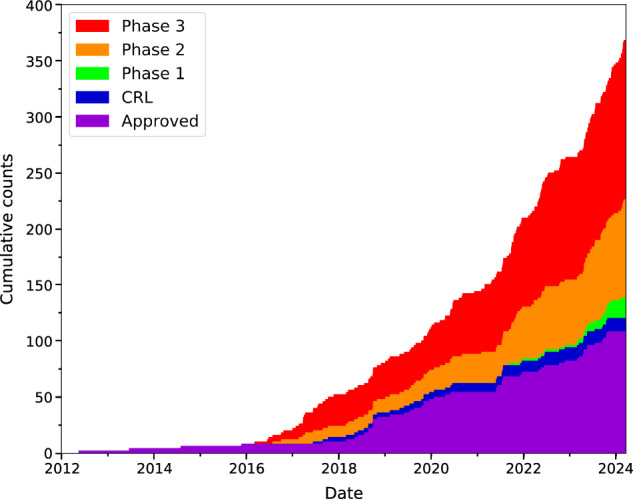
Cumulative number of antimicrobial treatments since 2012 and their respective clinical stage from publicly listed organisations. Stages in order (bottom to top): Approved (purple), CRL Complete Response Letter (blue), Phase 1 (green), Phase 2 (orange), and Phase 3 (red). Figure prepared using Matplotlib an open source python package and created with data in supplementary table 1 that was sourced from https://www.biopharmcatalyst.com/.

A review of all the reported antimicrobial treatment clinical trial data on www.biopharmacatlyst.com reveals that the number of antimicrobial treatments in the last decade has been on the rise (Fig. 1). However, there has also been a simultaneous increase in the total number of products receiving non-approval such as the complete response letters (CRL, Fig. 1) from the FDA. As of 2024, the number of antimicrobials undergoing clinical trials has nearly doubled compared with that of approved products, indicating increasing activity in this field. There are over 40 antimicrobials in interventional clinical trials, including the use of ‘non-traditional agents’ such as NAMs^18^. In Australia, we are aware of only two lipid NAM-based clinical trials, which are in Phase III, to investigate liposomal treatments for Mycobacterium (ALIS trial currently recruiting) and *Pseudomonas aeruginosa* infections (trial conducted in 2020) by Insmed Incorporated (Supplementary Table 1).

**Fig. 2:**
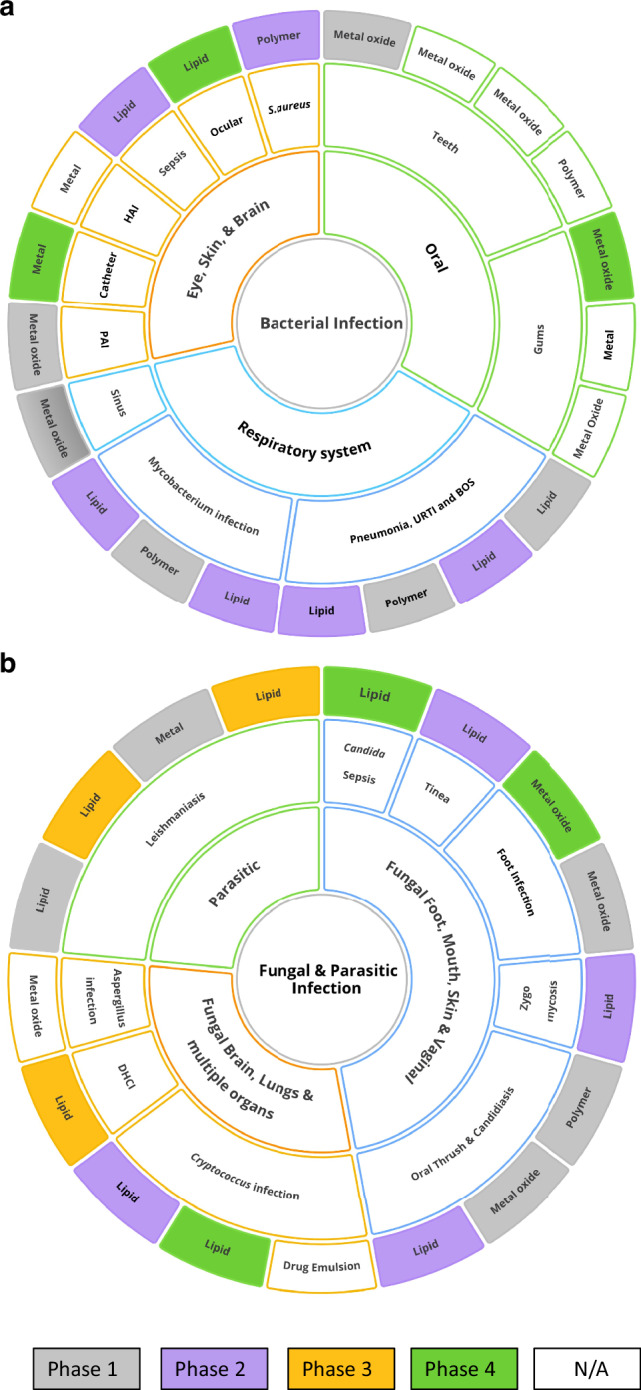
Schematic representation of Nano-antimicrobial based clinical trials. **a** Treatment for various bacterial infections. Wherein, BOS Bronchiolitis Obliterans Syndrome, PAI Pseudomonas aeruginosa Infection, URTI Upper respiratory tract infection, and HAI healthcare-associated infections (Nosocomial). **b** Treatment for various Fungal and Parasitic infections. Wherein, DHCI Disseminated Histoplasma Capsulatum Infection. Figure prepared using Ayoa Ultimate software.

We reviewed the global nano-antimicrobial-based clinical trials (listed on https://www.clinicaltrials.gov/) over the last 6 years, we have found 46 trials conducted or planned that specifically use NAMs to treat microbial infection (Fig. 2 and Supplementary Table 2). The increase in NAMs in clinical settings parallels the rise in conventional antimicrobial treatments, with the three-year average number of trials growing from just two between 1997 and 2000 to 12 trials from 2021 to 2024. However, a significant portion of these trials are in the Phase I stage (30%) and have relatively small participant numbers, averaging fewer than 100. Moreover, out of these 46 trials, only nine have posted results, and amongst them, five are accompanied by publications. This makes it challenging to demonstrate the statistical significance of NAMs’ potential benefits evidenced in clinical studies. The main clinically used materials to construct NAMs are lipid (liposomes and nanocochleates) and metal/metal oxide based (mainly silver, gold, iron, copper, and zinc), with a small number of polymers such as chitosan and poly(lactic-co-glycolic acid) polymers and peptide-based NAMs (Fig. 2 and Supplementary Table 2). Among the various nanocarriers available, lipid-based liposomes with cell membrane-like structures are a highly promising tool for antimicrobial drug delivery. Composed of amphiphilic lipids that form bilayers, liposomes can encapsulate hydrophilic drugs in their aqueous core and hydrophobic drugs within their lipid bilayer. This architecture not only provides a high drug-loading capacity but also enables liposomes to fuse with bacterial membranes, allowing for direct and potent delivery of antimicrobials into cells. Liposomes can be customised with different surface charges—neutral using phosphatidylcholine or lecithin, cationic with DOTAP(1,2-Dioleoyl-3-trimethylammonium propane), anionic with DOPS (1,2-dioleoyl-sn-glycero-3-phospholyserine), or zwitterionic using phosphatidylethanolamine—depending on the required interaction with microbial surfaces. The incorporation of PEG-lipids further enhances liposome stability, stealth characteristics, and biofilm penetration. With their fusogenic properties, tuneable surface charges, and ability to evade the immune system, liposomes stand out as one of the most promising nanocarriers for delivering antimicrobial agents^16,37^.

The list of target pathogens is almost evenly split between bacterial and fungal with a small number of parasitic diseases being addressed including cutaneous and disseminated Leishmaniasis (See Fig. 2 and Supplementary Table 2). The classes of microbes targeted and the NAMs being trialled are evolving rapidly as new technologies emerge. It is critically important that NAMs are scalable, commercially viable, and can be reproducibly manufactured within a regulated facility, which is discussed in the next section.

## Evidence requirements and Australian regulatory needs

NAMs are increasingly attractive as they may confer a benefit over molecular-based drugs^21,44^. From a regulatory perspective, NAMs are typically not required to be ‘better’ than standard antibiotic products; instead, they have the requirement of being ‘non-inferior’. In Australia, the TGA reviews and decides on applications for products that contain nanoparticles on a case-by-case basis. The US FDA has provided some guidance for developers of nanotechnology products, and the views of other major regulators are likely to influence the TGA’s future advice^7,45^. However, the TGA will continue to apply the legislative framework for assessing products in the Therapeutic Goods Act (1990), which requires products to demonstrate quality, safety, and efficacy in their intended use before receiving marketing approval. Nano-formulations of existing medicines (e.g. antibiotics) are new therapeutic goods under the Act, although reduced data requirements apply if a formulation is identical to one already registered by TGA. However, given that many parameters dictating the in vivo properties of NAMs are distributions of properties, rather than defined values, the measure of whether such formulations are identical requires careful assessment and development of well-defined tolerances.

For pre-clinical data, the TGA requires a demonstration of the reliability of engineering NAMs, including consistency and stability of particle size and/or stability of drug encapsulation, as is the case with lipid-based SARS-COV-2 mRNA vaccines. Data regarding the pharmacokinetics of particles in animal or human subjects may be required for novel nano-formulations, although the TGA can leverage its experience with established technologies (e.g., nanospheres and lipid nanoparticles) to reduce the data requirements for new applicants. Clinical trial data would need to demonstrate to the TGA the therapeutic claims and benefits of nanoparticle-based drugs compared with free drugs before they could be used as a basis for marketing approval.

The TGA can designate products for “Provisional” registration, which allows the regulator to approve a product based on preliminary data, provided confirmatory trials are conducted in the post-approval period. This expedited pathway is designed to allow early access to products that have a significant improvement in safety and/or efficacy over existing therapies. NAMs could potentially meet the criteria for provisional approval if the benefits of the nano-scale formulation were evident in early data. Developers of NAMs are encouraged to consult TGA early in the design of products, clinical trials, or regulatory dossiers to allow any issues with NAMs to be identified, and regulatory options to be determined.

There is a need to consistently characterise NAM formulations and improve guidelines^21^. This includes the formulation’s composition, purity, drug loading and entrapment efficiency, size, morphology, colloidal stability, structure, surface integrity, chemistry, surface charge, solubility, sterility, and drug release properties. In contrast to well-defined small molecule or protein therapeutics, these NAM formulations will have a distribution of particles, which display a range for each of these physicochemical properties rather than a defined value^17,21,46,47^. Therefore, the manufacturing and biological ramifications must be considered in this context^41,48^. Additional testing encompasses the biocompatibility and haemolytic properties, cytotoxicity in appropriate cell lines, immune response in animal models, endotoxin contamination testing, oxidative stress measurements in appropriate cell lines. Other indications include compliance with Good Manufacturing Practice (GMP), manufacturing process controls, batch analyses methods, control of all excipient materials, including understanding residual solvent or heavy metal levels, impurity levels and concentrations of all excipients, process validation, use of reference standards, and closed container systems^44^. Additional information on immunogenic properties, the interaction of NAMs with appropriate biological tissues, organs, and media, biodistribution, clearance, and comparison to appropriate reference materials is also key.

## Nanoantibiotic commercialisation and reimbursement opportunities

We know that the solutions to combat AMR are many and varied, but the development of safe and effective antibiotics is a public good. Australia could benefit from greater infrastructure, investment, and resources to improve ex vivo models for testing and screening of NAMs as well as clinical trial capacity. It is also a risk for countries to not have useful antibiotics. Repurposing old drugs in new NAMs and the use of combination therapies targeting two or more mechanisms provide some pathways forward^49^. There are opportunities to develop new incentives to reimburse research and development in this field, as antibiotics are low-cost medicines, with new ones used sparingly. Phase IV trials at the point that NAM treatments are used widely in the community are critical, therefore incentives could also assist in late-stage clinical trials. In Australia, for new drugs to be included in the Pharmaceutical Benefits Scheme (PBS), they require a committee of healthcare professionals and economists to determine whether the drugs offer value for money. For new antimicrobials, this may require a future shift away from the standard value assessment associated with cost per quality-adjusted life years towards a broader definition of value that considers the wider societal value and the concept of antimicrobials as a common good.

There are several interesting developments in the reimbursement space to manage the AMR market failure. New antimicrobial subscription models have been developed where governments would pay pharmaceutical companies annual fees for unfettered access to certain antibiotics^22^. Advanced market commitment models are also being considered for the repurposing of drugs and the development of new diagnostics that may help curb AMR. In the USA, this includes the CARB-X, and the creation of incentives via the Pioneering Antimicrobial Subscriptions to End Upsurging Resistance (PASTEUR), the Developing an Innovative Strategy for Antimicrobial-Resistant Microorganisms (DISARM) and the Generating Antibiotic Incentives Now (GAIN) Acts^50^. Governments globally are considering Health Technology Assessment fee exemptions (the full pathway from registration to market and funded access and maintenance charges) for products that address AMR and pathogens of concern.

## Future perspectives

There are numerous clinical and regulatory considerations that researchers need to contemplate before embarking on the development of NAM-based methods to treat infections. By understanding and addressing the challenges relating to the ‘nanoparticulate nature’ of these emerging therapies, and appreciating the physiology of the individual patient, further progress in this field can be made. As NAMs become increasingly important as antimicrobials, appropriate stewardship of such agents must be established to minimize the resistance to NAMs. Several resistance mechanisms for metal and carbon-based NAMs with inherent antimicrobial properties have been reported, for example, in soil microbes close to industrial facilities as well as in clinical pathogens^41,51–54^. Given the ever-increasing market for nanotechnology products, there are calls for greater regulation around their stewardship and development of mitigation and remediation strategies for minimising the prevalence of AMR genes in contaminated environments^51^. To this end, the UN and WHO have released guidance on waste management for the manufacturing of antibiotics (WHO, 2024). There are opportunities in the clinical regulatory landscape to take the inherent heterogeneity of nanomaterials into greater consideration. For example, this is significant when considering the approval of future generic NAM formulations, where there will be a need to consider acceptable tolerances for benchmarking similarity between NAMs. Given that the manufacturing process has a greater influence over the size, shape, surface, colloidal stability, and composition of NAMs compared with small molecule drugs, defining a regulatory pathway specific to nanomaterial therapeutics will become increasingly critical.

Beyond these aspects, improvements in NAM technologies may be achieved through greener and more sustainable synthetic production, the design of degradable systems that reduce their environmental persistence^55,56^, and the development of bespoke systems for personalised medicine^57^ Furthermore, better representation of minority populations is required when designing clinical trials for investigating these agents, which is particularly important in the Australian context, as well as globally^20^. We hope this article provides insights and guidance for developing new antimicrobial nanoparticles, and new formulations of existing antimicrobials and repurposed medicines. Ultimately, innovation and global collaboration are essential if we are to survive the AMR threat heading our way.

## Supplementary information

Supplementary information

## References

[1] Shi, Z. et al. A comprehensive overview of the antibiotics approved in the last two decades: retrospects and prospects. *Molecules* **28**, 1762 (2023). 36838752 10.3390/molecules28041762 PMC9962477

[2] Chahine, E. B., Dougherty, J. A., Thornby, K. A. & Guirguis, E. H. Antibiotic approvals in the last decade: are we keeping up with resistance? *Ann. Pharmacother.* **56**, 441–462 (2022). 34259076 10.1177/10600280211031390

[3] Butler, M. S., Henderson, I. R., Capon, R. J. & Blaskovich, M. A. T. Antibiotics in the clinical pipeline as of December 2022. *J. Antibiot. (Tokyo)* **76**, 431–473 (2023). 37291465 10.1038/s41429-023-00629-8 PMC10248350

[4] Collaborators, A. R. Global burden of bacterial antimicrobial resistance in 2019: a systematic analysis. *Lancet* **399**, 629–655 (2022). 35065702 10.1016/S0140-6736(21)02724-0 PMC8841637

[5] Butler, M. S. et al. Analysis of the clinical pipeline of treatments for drug-resistant bacterial infections: despite progress, more action is needed. *Antimicrob. Agents Chemother.* **66**, e0199121 (2022). 35007139 10.1128/aac.01991-21 PMC8923189

[6] Guillen, M. N., Li, C. R., Rosener, B. & Mitchell, A. Antibacterial activity of nonantibiotics is orthogonal to standard antibiotics. *Science* **384**, 93–100 (2024). 38484036 10.1126/science.adk7368 PMC12055234

[7] FDA. (ed. U.S. Department of Health and Human Services) 1–14 (Food and Drug Administration, Silver Spring, MD, USA, 2014).

[8] Dizaj, S. M., Lotfipour, F., Barzegar-Jalali, M., Zarrintan, M. H. & Adibkia, K. Antimicrobial activity of the metals and metal oxide nanoparticles. *Mater. Sci. Eng. C. Mater. Biol. Appl.* **44**, 278–284 (2014). 25280707 10.1016/j.msec.2014.08.031

[9] Makabenta, J. M. V. et al. Nanomaterial-based therapeutics for antibiotic-resistant bacterial infections. *Nat. Rev. Microbiol.* **19**, 23–36 (2021). 32814862 10.1038/s41579-020-0420-1 PMC8559572

[10] Aflakian, F. et al. Nanoparticles-based therapeutics for the management of bacterial infections: a special emphasis on FDA approved products and clinical trials. *Eur. J. Pharm. Sci.* **188**, 106515 (2023). 37402428 10.1016/j.ejps.2023.106515

[11] Paterson, D. L. Antibacterial agents active against gram negative bacilli in phase I, II, or III clinical trials. *Expert Opin. Investig. Drugs* **33**, 371–387 (2024). 38445383 10.1080/13543784.2024.2326028

[12] Ribeiro, A. I., Dias, A. M. & Zille, A. Synergistic effects between metal nanoparticles and commercial antimicrobial agents: a review. *ACS Appl. Nano Mater.* **5**, 3030–3064 (2022). 36568315 10.1021/acsanm.1c03891 PMC9773423

[13] Sanchez-Lopez, E. et al. Metal-based nanoparticles as antimicrobial agents: an overview. *Nanomaterials (Basel)* **10**, 292 (2020). 32050443 10.3390/nano10020292 PMC7075170

[14] Mondal, S. K., Chakraborty, S., Manna, S. & Mandal, S. M. Antimicrobial nanoparticles: current landscape and future challenges. *RSC Pharmaceutics* **1**, 388–402 (2024).

[15] Moradialvand, M., Asri, N., Jahdkaran, M., Beladi, M. & Houri, H. Advancements in nanoparticle-based strategies for enhanced antibacterial interventions. *Cell Biochem. Biophys.* **82**, 3071–3090 (2024). 39023679 10.1007/s12013-024-01428-0

[16] Thorn, C. R., Thomas, N., Boyd, B. J. & Prestidge, C. A. Nano-fats for bugs: the benefits of lipid nanoparticles for antimicrobial therapy. *Drug Deliv. Transl. Res.* **11**, 1598–1624 (2021). 33675007 10.1007/s13346-021-00921-w

[17] Ardal, C. et al. Antibiotic development - economic, regulatory and societal challenges. *Nat. Rev. Microbiol* **18**, 267–274 (2020). 31745330 10.1038/s41579-019-0293-3

[18] Mcdonnell, A., Dissanayake, R., Klemperer, K., Toxvaerd, F. & Sharland, M. *The Economics of Antibiotic Resistance, CGD Working Paper, 682* (2024).

[19] Piddock, L. J. V. et al. Advancing global antibiotic research, development and access. *Nat. Med.* **30**, 2432–2443 (2024). 39227444 10.1038/s41591-024-03218-w

[20] Umaefulam, V., Kleissen, T. & Barnabe, C. The representation of Indigenous peoples in chronic disease clinical trials in Australia, Canada, New Zealand, and the United States. *Clin. Trials* **19**, 22–32 (2022). 34991361 10.1177/17407745211069153 PMC8847750

[21] Dri, D. A., Rinaldi, F., Carafa, M. & Marianecci, C. Nanomedicines and nanocarriers in clinical trials: surfing through regulatory requirements and physico-chemical critical quality attributes. *Drug Deliv. Transl. Res.* **13**, 757–769 (2023). 36450964 10.1007/s13346-022-01262-y PMC9713170

[22] Berman, D. et al. Global access to existing and future antimicrobials and diagnostics: antimicrobial subscription and pooled procurement. *Lancet Glob. Health* **10**, e293–e297 (2022). 34914900 10.1016/S2214-109X(21)00463-0 PMC8765761

[23] Anderson, M. et al. Challenges and opportunities for incentivising antibiotic research and development in Europe. *Lancet Reg. Health Eur.* **33**, 100705 (2023). 37546576 10.1016/j.lanepe.2023.100705 PMC10403717

[24] Kirtane, A. R. et al. Nanotechnology approaches for global infectious diseases. *Nat. Nanotechnol.* **16**, 369–384 (2021). 33753915 10.1038/s41565-021-00866-8

[25] Mba, I. E. & Nweze, E. I. Nanoparticles as therapeutic options for treating multidrug-resistant bacteria: research progress, challenges, and prospects. *World J. Microbiol. Biotechnol.* **37**, 108 (2021). 34046779 10.1007/s11274-021-03070-x PMC8159659

[26] Golia, A., Mahmood, B. R., Fundora, Y., Thornby, K. A. & Chahine, E. B. Amikacin liposome inhalation suspension for mycobacterium avium complex lung disease. *Sr. Care Pharm.* **35**, 162–170 (2020). 32192565 10.4140/TCP.n.2020.162

[27] Rubino, C. M. et al. Correction to: population pharmacokinetic evaluation of amikacin liposome inhalation suspension in patients with treatment‑refractory nontuberculous mycobacterial lung disease. *Eur. J. Drug Metab. Pharmacokinet.* **46**, 573–574 (2021). 33595792 10.1007/s13318-020-00669-7 PMC7935831

[28] Shirley, M. Amikacin liposome inhalation suspension: a review in mycobacterium avium complex lung disease. *Drugs* **79**, 555–562 (2019). 30877642 10.1007/s40265-019-01095-z PMC6445814

[29] Zhang, J. et al. Amikacin liposome inhalation suspension (ALIS) penetrates non-tuberculous mycobacterial biofilms and enhances amikacin uptake into macrophages. *Front. Microbiol.* **9**, 915 (2018). 29867826 10.3389/fmicb.2018.00915 PMC5964161

[30] Fielding, R. M., Singer, A. W., Wang, L. H., Babbar, S. & Guo, L. S. Relationship of pharmacokinetics and drug distribution in tissue to increased safety of amphotericin B colloidal dispersion in dogs. *Antimicrob. Agents Chemother.* **36**, 299–307 (1992). 1605595 10.1128/aac.36.2.299 PMC188430

[31] Liu, Y. et al. Analytical method development and comparability study for AmBisome(R) and generic Amphotericin B liposomal products. *Eur. J. Pharm. Biopharm.* **157**, 241–249 (2020). 32980448 10.1016/j.ejpb.2020.09.008

[32] Olson, J. A. et al. Toxicity and efficacy differences between liposomal amphotericin B formulations in uninfected and Aspergillus fumigatus infected mice. *Med. Mycol.* **53**, 107–118 (2015). 25550388 10.1093/mmy/myu070

[33] Rivnay, B. et al. Critical process parameters in manufacturing of liposomal formulations of amphotericin B. *Int. J. Pharm.* **565**, 447–457 (2019). 31071418 10.1016/j.ijpharm.2019.04.052

[34] Shaikh, S. et al. Mechanistic insights into the antimicrobial actions of metallic nanoparticles and their implications for multidrug resistance. *Int. J. Mol. Sci.* **20**, 2468 (2019). 31109079 10.3390/ijms20102468 PMC6566786

[35] Chen, Y. et al. Nanomaterials against intracellular bacterial infection: from drug delivery to intrinsic biofunction. *Front. Bioeng. Biotechnol.* **11**, 1197974 (2023). 37180049 10.3389/fbioe.2023.1197974 PMC10174311

[36] Panthi, V. K., Fairfull-Smith, K. E. & Islam, N. Liposomal drug delivery strategies to eradicate bacterial biofilms: challenges, recent advances, and future perspectives. *Int. J. Pharm.* **655**, 124046 (2024). 38554739 10.1016/j.ijpharm.2024.124046

[37] Wang, D. Y., van der Mei, H. C., Ren, Y., Busscher, H. J. & Shi, L. Lipid-based antimicrobial delivery-systems for the treatment of bacterial infections. *Front. Chem.* **7**, 872 (2019). 31998680 10.3389/fchem.2019.00872 PMC6965326

[38] Kumar, L. et al. Advances in nanotechnology for biofilm inhibition. *ACS Omega* **8**, 21391–21409 (2023). 37360468 10.1021/acsomega.3c02239 PMC10286099

[39] Baptista, P. V. et al. Nano-strategies to fight multidrug resistant bacteria-“a battle of the titans. *Front. Microbiol.* **9**, 1441 (2018). 30013539 10.3389/fmicb.2018.01441 PMC6036605

[40] Blackman, L. D., Qu, Y., Cass, P. & Locock, K. E. S. Approaches for the inhibition and elimination of microbial biofilms using macromolecular agents. *Chem. Soc. Rev.* **50**, 1587–1616 (2021). 33403373 10.1039/d0cs00986e

[41] Blackman, L. D., Sutherland, T. D., De Barro, P. J., Thissen, H. & Locock, K. E. S. Addressing a future pandemic: how can non-biological complex drugs prepare us for antimicrobial resistance threats? *Mater. Horiz.* **9**, 2076–2096 (2022). 35703580 10.1039/d2mh00254j

[42] Luo, L., Huang, W., Zhang, J., Yu, Y. & Sun, T. L. Metal-based nanoparticles as antimicrobial agents: a review. *ACS Appl. Nano Mater.* **7**, 2529–2545 (2024).

[43] Pham, P., Oliver, S. & Boyer, C. Design of antimicrobial polymers. *Macromol. Chem. Phys.* **224**, 2200226 (2023).

[44] Dri, D. A. et al. Critical analysis and quality assessment of nanomedicines and nanocarriers in clinical trials: three years of activity at the clinical trials office. *Pharmaceutics* **14**, 1438 (2022). 35890333 10.3390/pharmaceutics14071438 PMC9318126

[45] Liposome Drug Products Chemistry, Manufacturing, and Controls; Human Pharmacokinetics and Bioavailability; and Labeling Documentation. Guidance for Industry FDA, 2018. http://www.fda.gov/Drugs/GuidanceComplianceRegulatoryInformation/Guidances/default.htm.

[46] Halamoda-Kenzaoui, B., Holzwarth, U., Roebben, G., Bogni, A. & Bremer-Hoffmann, S. Mapping of the available standards against the regulatory needs for nanomedicines. *Wiley Interdiscip. Rev. Nanomed. Nanobiotechnol.* **11**, e1531 (2019). 29923692 10.1002/wnan.1531 PMC6585614

[47] Ramos, T. I. et al. The Hitchhiker’s guide to human therapeutic nanoparticle development. *Pharmaceutics* **14**, 247 (2022). 35213980 10.3390/pharmaceutics14020247 PMC8879439

[48] Faria, M. et al. Minimum information reporting in bio-nano experimental literature. *Nat. Nanotechnol.* **13**, 777–785 (2018). 30190620 10.1038/s41565-018-0246-4 PMC6150419

[49] Lai, X. et al. Polysaccharide-targeting lipid nanoparticles to kill gram-negative bacteria. *Small* **20**, e2305052 (2024). 37798622 10.1002/smll.202305052

[50] Gregory, E. & Martin, C. The intersection of antimicrobial stewardship, the pharmaceutical industry, and the federal legislature. *Open Forum Infect. Dis.* **9**, ofac404 (2022). 36046701 10.1093/ofid/ofac404 PMC9423378

[51] Fang, Q. & Pan, X. A systematic review of antibiotic resistance driven by metal-based nanoparticles: Mechanisms and a call for risk mitigation. *Sci. Total Environ.* **916**, 170080 (2024). 38220012 10.1016/j.scitotenv.2024.170080

[52] McNeilly, O., Mann, R., Hamidian, M. & Gunawan, C. Emerging concern for silver nanoparticle resistance in acinetobacter baumannii and other bacteria. *Front. Microbiol.* **12**, 652863 (2021). 33936010 10.3389/fmicb.2021.652863 PMC8085274

[53] Zhang, C., Sun, R. & Xia, T. Adaption/resistance to antimicrobial nanoparticles: will it be a problem? *Nano Today* **34**, 100909 (2020).

[54] Nino-Martinez, N., Salas Orozco, M. F., Martinez-Castanon, G.-A., Torres Mendez, F. & Ruiz, F. Molecular mechanisms of bacterial resistance to metal and metal oxide nanoparticles. *Int. J. Mol. Sci.* **20**, 2808 (2019). 31181755 10.3390/ijms20112808 PMC6600416

[55] Landis, R. F. et al. Biodegradable nanocomposite antimicrobials for the eradication of multidrug-resistant bacterial biofilms without accumulated resistance. *J. Am. Chem. Soc.* **140**, 6176–6182 (2018). 29709168 10.1021/jacs.8b03575 PMC6044909

[56] Su, S. & Kang, P. M. Systemic review of biodegradable nanomaterials in nanomedicine. *Nanomaterials (Basel)* **10**, 656 (2020). 32244653 10.3390/nano10040656 PMC7221794

[57] Mitchell, M. J. et al. Engineering precision nanoparticles for drug delivery. *Nat. Rev. Drug Discov.* **20**, 101–124 (2021). 33277608 10.1038/s41573-020-0090-8 PMC7717100

