# Supplementary material for: An Australian perspective on clinical, economic and regulatory considerations in emerging nanoparticle therapies for infections
Source: NPJ Antimicrob Resist. 2025 Feb 18;3:9. doi: 10.1038/s44259-024-00070-3 (PMC11836273; doi:10.1038/s44259-024-00070-3)
Supplement: Supplementary file 1 — Supplementary information [file 44259_2024_70_MOESM1_ESM.pdf]

Table SI 1: Antimicrobial clinical trial pipeline data obtained from www.biopharmcatalyst.com

CRL = Complete Response Letter, IND = Investigational New Drug application, NDA = New Drug Application, PDUFA = Prescription Drug User Free Act.

Stock	Drug / treatment	Target	Stage	Date
ABBV	DALVANCE (dalbavancin)	Acute bacterial skin and skin structure infections (ABSSSI) - paediatric	Approved	23/07/2021
AMRX	FYLNETRA (pegfilgrastim-pbbk)	Neulasta biosimilar	Approved	27/05/2022
ANIP	Rifabutin Capsules	*Mycobacterium avium* complex	Approved	21/12/2021
ANIP	Nitrofurantoin Oral Suspension USP - (Furadantin generic)	Urinary tract infections	Approved	24/04/2023
APLIF	LIKMEZ (ATI-1501) - (liquid oral reformulation)	Parasitic and anaerobic bacterial infections	Approved	25/09/2023
BHC	PLENVU (NER1006)	Bowel cleansing	Approved	5/07/2018
BHC	ALTRENO1 (IDP-121)	Acne	Approved	24/08/2018
BHC	ARAZLO (tazarotene)	Acne	Approved	19/12/2019
BHC	CABTREO	Acne vulgaris	Approved	20/10/2023
CDTX	REZZAYO (rezafungin injection)	Candidemia	Approved	22/03/2023
CHRS	CHS-1701	Pegfilgrastim biosimilar	Approved	11/02/2018
CHRS	CHS-1701	Pegfilgrastim biosimilar	Approved	3/06/2023
DARE	DARE-BV1	Bacterial vaginosis	Approved	12/07/2021
EBS	BioThrax	Anthrax vaccine	Approved	17/05/2012
EBS	BioThrax	Anthrax vaccine adsorbed	Approved	24/11/2015
EBS	CYFENDUS (AV7909)	Anthrax vaccine	Approved	21/07/2023
GSK	KRINTAFEL (Tafenoquine)	Malaria	Approved	23/07/2018
GSK	Menveo (Meningococcal Oligosaccharide Diptheria CRM197 Conjugate Vaccine)	Meningococcal disease	Approved	17/10/2022
INSM	ARIKAYCE	Nontuberculous mycobacterial (NTM) lung disease	Approved	28/09/2018

INVA	XACDURO (sulbactam for injection; durlobactam for injection)	Hospital-acquired bacterial pneumonia and ventilator-associated bacterial pneumonia (HABP/VABP) caused by susceptible isolates of *Acinetobacter baumannii*-calcoaceticus complex (Acinetobacter)	Approved	24/05/2023
LGND	VAXNEUVANCE	Pneumococcal disease	Approved	16/07/2021
LJPC	XERAVA (eravacycline)	cIAI (complicated intra-abdominal infections)	Approved	27/08/2018
MCRB	Vowst (SER-109)	Recurrent *Clostridium difficile* infection	Approved	26/04/2023
MRK	ZERBAXA(ceftolozane and tazobactam)	Hospital-acquired bacterial pneumonia (HABP)	Approved	6/03/2019
MRK	PRIMAXIN (imipenem and cilastatin)	Complicated urinary tract infections (cUTI) and Complicated intra-abdominal infections (cIAI)	Approved	17/07/2019
MRK	DIFICID (fidaxomicin)	*Clostridium difficile* infections (CDI)	Approved	27/01/2020
MRK	RECARBRIO (imipenem, cilastatin, and relebactam)	Hospital-Acquired and Ventilator-Associated Bacterial Pneumonia (HABP/VABP)	Approved	6/04/2020
MRK	VAXNEUVANCE	Pneumococcal disease	Approved	16/07/2021
MRK	VAXNEUVANCE (V114-029) - (PNEU-PED)	Pneumococcal vaccine in children	Approved	22/06/2022
NBRV	XENLETA (lefamulin)	Moderate to severe community acquired bacterial pneumonia (CABP)	Approved	19/08/2019
NVS	ORBACTIV (Oritavancin)	ABSSSI	Approved	8/06/2014
NVS	VABOMERE (meropenem and vaborbactam)	Complicated urinary tract infections (cUTI)	Approved	29/08/2017
NVS	EGATEN (triclabendazole)	Fascioliasis	Approved	13/02/2019
NVS	Biosimilar pegfilgrastim	Pegfilgrastim biosimilar	Approved	11/05/2019
OTIC	OTIPRIO	Acute otitis externa	Approved	3/02/2018
PFE	PREVNAR 20	Pneumococcal conjugate vaccine	Approved	6/08/2021
PFE	20vPnC	Pneumococcal disease	Approved	27/04/2023
PFE	PENBRAYA (MenABCWY)	Meningococcal vaccine, adolescents	Approved	20/10/2023
PHAT	VOQUEZNA	*H. pylori*	Approved	5/03/2022
PHAT	VOQUEZNA	*H. pylori*	Approved	30/10/2023

PRTK	NUZYRA (omadacycline)	Acute bacterial skin and skin structure infections (ABSSSI)	Approved	10/02/2018
PRTK	NUZYRA (omadacycline)	Community-acquired bacterial pneumonia (CABP)	Approved	10/02/2018
PRTK	Sarecycline	Severe acne vulgaris	Approved	10/02/2018
PRTK	NUZYRA (omadacycline) - oral	Acute bacterial skin and skin structure infections (ABSSSI)	Approved	6/01/2021
RDHL	TALICIA (RHB-105)	*H. pylori*	Approved	11/04/2019
RDHL	TALICIA (RHB-105)	*H. pylori*	Approved	18/09/2023
SCYX	BREXAFEMME (ibrexafungerp)	Vulvovaginal candidiasis (VVC)	Approved	6/02/2021
SCYX	BREXAFEMME (ibrexafungerp) - (CANDLE)	Recurrent vulvovaginal candidiasis (rVVC), vaginal yeast infections	Approved	12/01/2022
SLGL	Twyneo	Acne	Approved	27/07/2021
SNY	MenQuadfi	Meningococcal meningitis	Approved	24/04/2020
TBPH	VIBATIV (telavancin hydrochloride) for injection	Hospital-acquired and ventilator-associated bacterial pneumonia (HABP/VABP)	Approved	21/06/2013
VTRS	FULPHILA (Neulasta biosimilar)	Neulasta biosimilar	Approved	6/04/2018
VYNE	AMZEEQ (FMX101)	Acne	Approved	18/10/2019
ZLAB	XACDURO (sulbactam for injection; durlobactam for injection)	Hospital-acquired bacterial pneumonia and ventilator-associated bacterial pneumonia (HABP/VABP) caused by susceptible isolates of *Acinetobacter baumannii*-calcoaceticus complex (Acinetobacter)	Approved	24/05/2023
CHRS	CHS-1701	Pegfilgrastim biosimilar	CRL	6/12/2017
ITRM	Sulopenem etzadroxil-probenecid	Uncomplicated urinary tract infections (uUTI)	CRL	26/07/2021
NBRV	Contepo	Complicated urinary tract infections (cUTI)	CRL	30/04/2019
NBRV	Contepo	Complicated urinary tract infections (cUTI)	CRL	19/06/2020
SPRO	Tebipenem (SPR994)	Complicated urinary tract infections (cUTI)	CRL	27/06/2022
VTRS	FULPHILA (Neulasta biosimilar)	Neulasta biosimilar	CRL	10/10/2017

AUPH	AUR300	M2 Macrophage	IND-Enabling	15/02/2024
ITRM	Sulopenem etzadroxil-probenecid	Uncomplicated urinary tract infections (uUTI)	NDA Filing	7/01/2021
EBS	Anthrax Immune Globulin Intravenous (Human) [AIGIV]	Anthrax	PDUFA	25/03/2015
DERM	DFD-29-CD-006	Microbial flora, papulopustular rosacea	Phase 1	13/06/2023
JAGX	Mytesi (crofelemer)	Moderate-to-severe diarrhea including *Vibrio cholerae*	Phase 1	2/12/2024
RXRX	REC-3964	*Clostridium difficile* Infection	Phase 1	9/05/2023
ARMP	AP-PA02 - (SWARM-P.a.)	*Pseudomonas aeruginosa* Infections and Cystic Fibrosis	Phase 1/2	3/06/2023
MTNB	Oral amphotericin b (MAT2203) - (EnACT)	Cryptococcal meningitis	Phase 1/2	13/09/2021
PCVX	VAX-31	Invasive Pneumococcal Disease (IPD)	Phase 1/2	11/09/2023
PHGE	BX004	*Pseudomonas aeruginosa* in patients with Cystic Fibrosis (CF)	Phase 1/2	29/11/2023
CFRX	CF-370	Gram-negative infections	Phase 1a	16/10/2023
MCRB	SER-155	Gastrointestinal infections, bacteraemia and graft versus host disease (GvHD)	Phase 1b	5/09/2023
ANAB	Imsidolimab (ANB019) - (ACORN)	Acne	Phase 2	14/03/2022
ARDS	Aerucin (AR-105)	*Pseudomonas aeruginosa*	Phase 2	9/03/2019
CDTX	Rezafungin (CD101) - RADIANT	Vulvovaginal candidiasis (VVC)	Phase 2	21/02/2017
CDTX	Rezafungin (CD101) - (ReSTORE)	Candidemia	Phase 2	19/03/2018
CDTX	Rezafungin (CD101) - (STRIVE B)	Candidemia	Phase 2	29/07/2019
CFRX	Exebacase - (DISRUPT)	Serious infections caused by *Staphylococcus aureus* including MRSA	Phase 2	1/07/2019
CFRX	Exebacase - (DISRUPT)	Serious infections caused by *Staphylococcus aureus* including MRSA	Phase 2	10/04/2021
ENLV	Allocetra	Sepsis	Phase 2	21/02/2024
FNCH	CP101 - (PRISM3)	*Clostridium difficile* infection	Phase 2	25/10/2021

FNCH	CP101 - (PRISM-EXT)	*Clostridium difficile* infection	Phase 2	11/09/2021
FNCH	CP101 - (PRISM-EXT)	*Clostridium difficile* infection	Phase 2	6/01/2022
FNCH	CP101 - (PRISM3)	*Clostridium difficile* infection	Phase 2	6/01/2022
FNCH	CP101 - (PRISM-EXT)	*Clostridium difficile* infection	Phase 2	24/10/2022
MCRB	SER-109 - (ECOSPOR)	Recurrent *Clostridium difficile* infection	Phase 2	29/07/2016
NOVN	SB208	Fungal infections	Phase 2	4/12/2017
PALI	LB1148 - (China trial)	Bowel function following abdominal surgery	Phase 2	29/07/2021
PCVX	VAX-24	Pneumococcal conjugate vaccine	Phase 2	17/04/2023
PCVX	VAX-24	Pneumococcal disease in infants	Phase 2	3/04/2024
PFE	VLA15-221	Lyme disease	Phase 2	28/09/2021
PFE	VLA15-221	Lyme disease	Phase 2	4/02/2022
PFE	PF-06760805 (GBS6)	Streptococcus Vaccine	Phase 2	19/07/2023
PFE	VLA15-221	Lyme disease	Phase 2	9/07/2023
PHGE	BX001	Acne	Phase 2	18/10/2021
PHGE	BX001	Acne	Phase 2	15/11/2021
PRTC	VE303 - (CONSORTIUM)	Clostridioides difficile infection (CDI)	Phase 2	5/09/2023
SPRO	SPR720	Nontuberculous mycobacterial (NTM) pulmonary disease	Phase 2	2/05/2021
SVRA	Molgradex - OPTIMA	Nontuberculous mycobacteria (NTM)	Phase 2	3/12/2020
SXTP	Tafenoquine regimen of ARAKODA	Babesiosis	Phase 2	22/01/2024
TFFP	Voriconazole Inhalation Powder - (VORI)	Invasive pulmonary Aspergillosis (IPA)	Phase 2	19/12/2023
VALN	VLA15-221	Lyme disease	Phase 2	28/09/2021
VALN	VLA15-221	Lyme disease	Phase 2	4/02/2022
VALN	VLA15-221	Lyme disease	Phase 2	9/07/2023
VYNE	FCD105	Acne	Phase 2	6/02/2020
XENE	XEN801	Acne	Phase 2	24/03/2017
EGRX	CAL02	Severe Community-Acquired Bacterial Pneumonia (SCABP)	Phase 2/3	14/06/2023
ACXP	Ibezapolstat	*Clostridium difficile* infection	Phase 2a	11/05/2021

LIPO	LP-10 (liposomal tacrolimus)	Haemorrhagic cystitis	Phase 2a	30/04/2023
SVRA	Molgradex - ENCORE	Nontuberculous mycobacterial (NTM) lung infection with cystic fibrosis (CF)	Phase 2a	9/03/2020
TARS	TP-05 - (Carpo)	Lyme disease	Phase 2a	22/02/2024
ACXP	Ibezapolstat	*Clostridium difficile* infection	Phase 2b	27/02/2024
DRMA	DMT310 - (STAR-1)	Acne	Phase 2b	27/06/2023
EVFM	PHEXXI (Amphora)	Prevention of urogenital *Chlamydia trachomatis* infection in women	Phase 2b	12/02/2019
PXMD	PAX-101 - (suramin)	Human African Trypanosomiasis (HAT)	Phase 2b	24/07/2023
TENX	Levosimendan	Septic shock	Phase 2b	10/05/2016
ARDS	AR-301 (tosatoxumab)	Ventilator associated pneumonia (VAP) / *Staphylococcus aureus*	Phase 3	20/06/2023
BHC	IDP-126	Acne	Phase 3	22/04/2021
CDTX	Rezafungin (CD101) - (ReSTORE)	Candidemia	Phase 3	14/12/2021
CFRX	Exebacase - (DISRUPT)	Serious infections caused by *Staphylococcus aureus* including MRSA	Phase 3	13/07/2022
EVFM	EVO100 - (EVOGUARD)	Chlamydia and gonorrhoea	Phase 3	10/12/2022
GSK	MenABCWY	Meningitis, meningococcal disease caused by serogroups A, B, C, W and Y	Phase 3	5/12/2023
GSK	Gepotidacin - (EAGLE 2/3)	Uncomplicated urinary tract infection (uUTI)	Phase 3	26/02/2024
INVA	SUL-DUR - (ATTACK)	*Acinetobacter baumannii* infections	Phase 3	18/10/2021
INVA	SUL-DUR - (ATTACK)	*Acinetobacter baumannii infections*	Phase 3	26/04/2022
INVA	SUL-DUR - (ATTACK)	*Acinetobacter baumannii* infections	Phase 3	17/05/2022
INVA	Zoliflodacin	Uncomplicated gonorrhoea	Phase 3	11/01/2023
ITRM	Sulopenem	Complicated intra-abdominal infections (cIAI)	Phase 3	12/10/2019
ITRM	Sulopenem	Complicated urinary tract infections (cUTI)	Phase 3	6/01/2020
ITRM	Sulopenem etzadroxil-probenecid	Uncomplicated urinary tract infections (uUTI)	Phase 3	29/06/2020
ITRM	Sulopenem etzadroxil-probenecid - (REASSURE)	Uncomplicated urinary tract infections (uUTI)	Phase 3	30/01/2024

LGND	VAXNEUVANCE	Pneumococcal disease	Phase 3	22/06/2020
LGND	V116 - (STRIDE-6)	Pneumococcal Conjugate Vaccine	Phase 3	27/07/2023
LGND	V116 - (STRIDE-3)	Pneumococcal conjugate vaccine	Phase 3	28/11/2023
LJPC	XERAVA (eravacycline)	cIAI (complicated intra-abdominal infections)	Phase 3	25/07/2017
MCRB	SER-109 - (ECOSPOR)	Recurrent *Clostridium difficile* infection	Phase 3	8/10/2020
MCRB	SER-109 - (ECOSPOR)	Recurrent *Clostridium difficile* infection	Phase 3	10/04/2021
MCRB	SER-109 - (ECOSPOR)	Recurrent *Clostridium difficile* infection	Phase 3	22/05/2022
MCRB	SER-109 - (ECOSPOR)	Recurrent *Clostridium difficile* infection	Phase 3	6/07/2022
MRK	ZERBAXA(ceftolozane and tazobactam)	Hospital-acquired bacterial pneumonia (HABP)	Phase 3	9/11/2018
MRK	VAXNEUVANCE	Pneumococcal disease	Phase 3	22/06/2020
MRK	V114 (PNEU-DIRECTION)	Pneumococcal conjugate vaccine	Phase 3	20/05/2021
MRK	VAXNEUVANCE (V114-029) - (PNEU-PED)	Pneumococcal Vaccine in children	Phase 3	25/08/2021
MRK	V116 - (STRIDE-6)	Pneumococcal conjugate vaccine	Phase 3	27/07/2023
MRK	V116 - (STRIDE-3)	Pneumococcal conjugate vaccine	Phase 3	28/11/2023
MTNB	Oral amphotericin b (MAT2203) - (EnACT)	Cryptococcal meningitis	Phase 3	26/02/2024
NBRV	XENLETA (lefamulin)	Moderate to severe Community Acquired Bacterial Pneumonia (CABP)	Phase 3	18/09/2017
NKTR	Inhaled Amikacin Solution (BAY41-6551T)	Gram-negative Pneumonia (INHALE 1)	Phase 3	24/11/2017
NOVN	SB204	Acne vulgaris	Phase 3	27/01/2017
NVS	VABOMERE (meropenem and vaborbactam)	Complicated urinary tract infections (cUTI)	Phase 3	27/06/2016
OTIC	OTIPRIO	Acute otitis externa	Phase 3	1/05/2017
PFE	PF-06425090	*Clostridium difficile* infection	Phase 3	3/01/2022
PFE	Aztreonam-avibactam (ATM-AVI) - (REVISIT)	Complicated Intra-abdominal Infection and Hosptial Acquired Pneumonia	Phase 3	6/01/2023
PFE	Aztreonam-avibactam (ATM-AVI) - (ASSEMBLE)	Serious bacterial infection	Phase 3	6/01/2023
PFE	VLA15 - (VALOR)	Lyme disease vaccine for outdoor recreationists	Phase 3	12/04/2023

PHAT	VOQUEZNA	*H. pylori*	Phase 3	29/04/2021
PLXP	Locilex	Mild infections of diabetic foot ulcers	Phase 3	25/10/2016
PRTK	NUZYRA (omadacycline)	Acute bacterial skin and skin structure infections (ABSSSI)	Phase 3	16/06/2016
PRTK	Sarecycline	Severe acne vulgaris	Phase 3	27/03/2017
PRTK	NUZYRA (omadacycline)	Community-acquired bacterial pneumonia (CABP)	Phase 3	4/03/2017
PRTK	NUZYRA (omadacycline) - oral	Acute bacterial skin and skin structure infections (ABSSSI)	Phase 3	17/07/2017
PYPD	D-PLEX100 - (SHIELD I)	Abdominal (soft tissue) sternal surgical site infections	Phase 3	9/02/2022
PYPD	D-PLEX100 - (SHIELD II)	Surgical Site Infection	Phase 3	2/12/2024
RDHL	TALICIA (RHB-105)	*H. pylori*	Phase 3	3/08/2016
RDHL	BEKINDA	Gastroenteritis	Phase 3	14/06/2017
RDHL	TALICIA (RHB-105)	*H. pylori*	Phase 3	12/03/2018
SCYX	BREXAFEMME (ibrexafungerp) - (FURI)	Invasive candidiasis	Phase 3	30/01/2019
SCYX	BREXAFEMME (ibrexafungerp) - (FURI)	Invasive candidiasis	Phase 3	1/08/2020
SCYX	BREXAFEMME (ibrexafungerp)	Vulvovaginal candidiasis (VVC)	Phase 3	21/04/2020
SCYX	SCY-078 (oral) - (CARES)	*Candida auris*	Phase 3	3/02/2021
SCYX	BREXAFEMME (ibrexafungerp) - (FURI)	Invasive candidiasis	Phase 3	3/02/2021
SCYX	BREXAFEMME (ibrexafungerp) - (FURI)	Invasive candidiasis	Phase 3	29/09/2021
SCYX	BREXAFEMME (ibrexafungerp) - (CANDLE)	Recurrent Vulvovaginal candidiasis (rVVC), Vaginal yeast infections	Phase 3	2/10/2022
SCYX	BREXAFEMME (ibrexafungerp) - (FURI)	Invasive candidiasis	Phase 3	5/10/2022
SCYX	BREXAFEMME (ibrexafungerp) - (CANDLE)	Recurrent Vulvovaginal candidiasis (rVVC), Vaginal yeast infections	Phase 3	19/07/2022
SCYX	BREXAFEMME (ibrexafungerp) - (FURI)	Invasive candidiasis	Phase 3	24/10/2022
SLGL	Twyneo	Acne	Phase 3	30/12/2019
SMMT	Ridinilazole - (Ri-CoDIFy)	*Clostridium difficile* infection	Phase 3	20/12/2021

SMMT	Ridinilazole - (Ri-CoDIFy)	*Clostridium difficile* infection	Phase 3	20/10/2022
SPRO	Tebipenem (SPR994)	Complicated urinary tract infections (cUTI)	Phase 3	9/08/2020
SPRO	Tebipenem HBr (SPR994) - (PIVOT-PO)	Complicated urinary tract infection (cUTI), including acute pyelonephritis (AP)	Phase 3	1/02/2024
SVRA	AeroVanc - AVAIL	MRSA in cystic fibrosis	Phase 3	12/10/2020
VALN	VLA15 - (VALOR)	Lyme disease vaccine for outdoor recreationists	Phase 3	12/04/2023
VYNE	AMZEEQ (FMX101)	Acne	Phase 3	27/03/2017
ZLAB	SUL-DUR - (ATTACK)	*Acinetobacter baumannii* infections	Phase 3	18/10/2021
ZLAB	SUL-DUR - (ATTACK)	*Acinetobacter baumannii* infections	Phase 3	26/04/2022
ZLAB	SUL-DUR - (ATTACK)	*Acinetobacter baumannii* infections	Phase 3	17/05/2022
SCYX	SCY-247	Pulmonary mucormycosis	Preclinical	30/01/2024

Table SI 2: NAM based clinical trials obtained from https://www.clinicaltrials.gov/

Bacterial Infection									
No	Pathogen / Disease	Nanoparticle type	Nanoparticle composition	Active ingredients	Effected organ / Area	Clinical Stage	Results Posted	Enrolment	Trial Number
1	Apical Periodontitis	Metal oxide	Iron oxide	Ferumoxytol	Mouth/Teeth and Gums	4	N	44	NCT06089720
2	Gingivitis / Periodontitis	Metal	Gold	Pelargonium Graveolens Leaves Extract	Mouth/Teeth and Gums	n/a	N	60	NCT05816512
3	Tooth infection	Metal and metal oxide	Silver / Calcium hydroxide	Silver / Calcium hydroxide	Mouth/Teeth and Gums	n/a	N	22	NCT05681221
4	Gingival Inflammation	Metal oxide	Titanium dioxide	Titanium dioxide	Mouth/Teeth and Gums	n/a	N	26	NCT06051487
5	Teeth bacterial colonization	Metal oxide	Silver fluoride	Silver fluoride	Mouth/Teeth and Gums	3	Y[1]	50	NCT05221749
6	Teeth infection	Metal oxide	Zinc oxide	Zinc oxide	Mouth/Teeth and Gums	n/a	N	18	NCT05901961
7	Bacterial infections – oral	Polymer	PLGA / Chitosan	Ciprofloxacin	Mouth/Teeth and Gums	n/a	N	55	NCT05475444
8	Ulcerative Keratitis	Metal and metal oxide	Gold-Silver alloy / Copper oxide	Voriconazole	Eye	1	N	20	NCT05268718
9	Central venous catheter related infections	Metal	Silver	Silver	Brain	4	N	472	NCT00337714
10	Nosocomial infections	Metal	Silver / Copper	Silver / Copper	Multiple organ	n/a	N	100	NCT04775238
11	Ocular infections	Lipid	OZODROP® (ozonated vegetable oil, soy phospholipids)	OZODROP®	Eye	4	N	200	NCT04087733
12	Staphylococcus aureus	Polymer	PMX-30063	PMX-30063	Skin/Nose	2	N	215	NCT01211470
13	Chronic rhinosinusitis	Metal oxide	Silver	Silver	Nose and head	1	N	0	NCT03243201
14	Sepsis	Lipid	Smoflipid (Fish oil emulsion)	Smoflipid	Multiple organs	1&2	Y[2]	68	NCT03405870
15	Bronchiolitis obliterans syndrome	Lipid	Nonionic surfactants and phospholipids (exact details not disclosed)	Cyclosporine A	Lungs	2	Y[3]	6	NCT03656926
16	Upper respiratory tract infections	Polymer	Proprietary formulation not disclosed	PrEP-001	Lungs	1	Y[4]	20	NCT 03220048
17	Pneumonia – bacterial	Lipid	CAL02 (Cholesterol and sphingomyelin)	CAL02	Lungs	2	N	276	NCT02583373
18	Antibiotic resistant infection	Polymer	PLGA / chitosan	Ciprofloxacin	Lungs	1	N	55	NCT05475444
19	Lung Infections	Lipid	Mesenchymal Progenitor Cell-derived exosomes	MPCs-derived exosomes	Lungs	1&2	N	60	NCT04544215
20	Mycobacterium infections	Lipid	DPPC, cholesterol	Amikacin	Lungs	2	N	30	NCT02344004
21	Mycobacterium infections	Lipid	DPPC, cholesterol	Amikacin, Azithromycin, Ethambutol	Lungs	3	N	250	NCT04677569

22	Mycobacterium infections	Lipid	DPPC, cholesterol	Amikacin	Lungs	2	Y[5,6]	90	NCT03038178
23	Mycobacterium infections	Polymer	Glutathione-cyclodextrin	Glutathione-cyclodextrin	Lungs	1	N	30	NCT05926245
**Fungal and Parasitic Infection**									
No	Pathogen/Disease	Nanoparticle type	Nanoparticle composition	Active ingredients	Effected organ / Area	Clinical Stage	Results Posted	Enrolment	Trial Number
1	Candida fungal	Lipid	Ambisome® (Hydrogenated soy phosphatidyl-choline, Distearoyl-phosphatidyl glycerol, Cholesterol)	Amphotericin B	Foot, mouth, throat, gut, vagina	4	N	30	NCT00697944
2	Candidiasis	Lipid	Phosphatidyl-serine and Calcium	Amphotericin B	Foot, mouth, throat, gut, vagina	2	Y[7]	4	NCT02629419
3	Foot infection – fungal	Metal	Silver	Silver	Foot	1	N	30	NCT02629419
4	Candida infection	Metal oxide	Titanium dioxide	Titanium dioxide	Foot, mouth	1	N	20	NCT05901961
5	Oral thrush	Polymer	Chitosan	Miconazol	Mouth	1	N	80	NCT06072716
6	Zygomycosis	Lipid	Ambisome® (Hydrogenated soy phosphatidyl-choline, Distearoyl-phosphatidyl glycerol, Cholesterol)	Amphotericin B	Mouth/face	2	N	40	NCT00467883
7	Fungal foot infection	Metal oxide	Zinc oxide	Salicylic acid, benzoic acid (Whitfield solution)	Foot	4	N	84	NCT05901961
8	Tinea versicolor	Lipid	Cotton seed oil, Span80	Itraconazole	Skin/nose	2	N	30	NCT04110834
9	Pseudomonas aeruginosa infection	Lipid	DPPC, cholesterol	Amikacin	Multiple organ	3	Y[8]	302	(NCT01315678
10	Cryptococcal meningitis	Lipid	Proprietary formulation not disclosed	Amphotericin B	Multiple organs	4	N	40	NCT03945448
11	Cryptococcal meningitis	Drug emulsion	Cholesterol sulfate	Amphotericin B	Multiple organs	n/a	N	30	NCT00263315
12	Disseminated histoplasma capsulatum infection	Lipid	Ambisome® (Hydrogenated soy phosphatidyl-choline, Distearoyl-phosphatidyl glycerol, Cholesterol)	Amphotericin B	Multiple organs	3	N	254	NCT04431804
13	Aspergillosis	Lipid	Ambisome® (Hydrogenated soy phosphatidyl-choline, Distearoyl-phosphatidyl glycerol, Cholesterol)	Amphotericin B	Lungs	2&3	N	320	NCT06000514
14	Aspergillosis	Metal	Silver	Silver	Lungs	n/a	N	210	NCT01050777
15	Cutaneous leishmaniasis	Metal	Gold / Sm29 Protein	Gold / Sm29 Protein	Multiple organs	1&2	N	20	NCT03636659

16	Cutaneous leishmaniasis	Lipid	Soybean phosphatidyl-choline, Cholesterol , Propyl-paraben, Methyl-paraben, propylene glycol and vitamin E	Glucantime (Meglumine Antimoniate), Paromomycin	Multiple organs	1	N	30	NCT05471063
17	Visceral leishmaniasis	Lipid	Proprietary formulation not disclosed	Amphotericin B	Multiple organs	1	N	140	NCT02136030
18	Cryptococcal meningitis	Lipid	Phosphatidyl-serine and Calcium	Amphotericin B	Multiple organs	1&2	N	0	NCT00003315
19	Cryptococcal meningitis	Lipid	Ambisome® (Hydrogenated soy phosphatidyl-choline, Distearoyl-phosphatidyl glycerol, Cholesterol)	Amphotericin B, Fluconazole	Multiple organs	2&3	N	356	NCT02025491
20	Cryptococcal meningitis	Lipid	Proprietary formulation not disclosed	Amphotericin B	Multiple organs	n/a	N	84	NCT00386997
21	Infection	Lipid	Proprietary formulation not disclosed	Amphotericin B	Multiple organs	3	N	200	(NCT01315678
22	Disseminated leishmaniasis	Lipid	Proprietary formulation not disclosed	Amphotericin B	Multiple organs	3	N	20	NCT03945448
23	Fungal infection	Lipid	Ambisome® (Hydrogenated soy phosphatidyl-choline, Distearoyl-phosphatidyl glycerol, Cholesterol)	Amphotericin B	Multiple organs	4	N	20	NCT00263315

References

1 Ammar, N. *et al.* The antibacterial effect of nanosilver fluoride in relation to caries activity in primary teeth: a protocol for a randomised controlled clinical trial. *Trials* **23**, 558 (2022). https://doi.org:10.1186/s13063-022-06477-5

2 Guirgis, F. W. *et al.* Lipid intensive drug therapy for sepsis pilot: A Bayesian phase I clinical trial. *J Am Coll Emerg Physicians Open* **1**, 1332-1340 (2020). https://doi.org:10.1002/emp2.12237

3 Neurohr, C. *et al.* A Randomized controlled trial of liposomal cyclosporine A for inhalation in the prevention of bronchiolitis obliterans syndrome following lung transplantation. *Am J Transplant* **22**, 222-229 (2022). https://doi.org:10.1111/ajt.16858

4 Malcolm, B. A. *et al.* PrEP-001 Prophylactic effect against rhinovirus and influenza virus - results of 2 randomised trials. *Antiviral Res* **153**, 70-77 (2018). https://doi.org:10.1016/j.antiviral.2018.03.005

5 Rubino, C. M. *et al.* Population pharmacokinetic evaluation of Amikacin liposome inhalation suspension in patients with treatment-refractory nontuberculous mycobacterial lung disease. *Eur J Drug Metab Pharmacokinet* **46**, 277-287 (2021). https://doi.org:10.1007/s13318-020-00669-7

6 Olivier, K. N. *et al.* Randomised trial of liposomal Amikacin for inhalation in Nontuberculous Mycobacterial lung disease. *Am J Respir Crit Care Med* **195**, 814-823 (2017). https://doi.org:10.1164/rccm.201604-0700OC

7 Desai, J. V. *et al.* Efficacy of cochleated Amphotericin B in mouse and Human mucocutaneous *Candidiasis*. *Antimicrob Agents Chemother* **66**, e0030822 (2022). https://doi.org:10.1128/aac.00308-22

8 Bilton, D. *et al.* Amikacin liposome inhalation suspension for chronic *Pseudomonas aeruginosa* infection in cystic fibrosis. *J Cyst Fibros* **19**, 284-291 (2020). https://doi.org:10.1016/j.jcf.2019.08.001
